# 3-Chloro-6-(3,5-dimethyl-1*H*-pyrazol-1-yl)pyridazine

**DOI:** 10.1107/S1600536810034756

**Published:** 2010-09-04

**Authors:** Abdul Qayyum Ather, M. Nawaz Tahir, Misbahul Ain Khan, Muhammad Makshoof Athar, Eliana Aparecida Silicz Bueno

**Affiliations:** a Department of Chemistry, Islamia University, Bahawalpur, Pakistan; b Applied Chemistry Research Center, PCSIR Laboratories complex, Lahore 54600, Pakistan; c Department of Physics, University of Sargodha, Sargodha, Pakistan; d Institute of Chemistry, University of the Punjab, Lahore, Pakistan; e Instituto de Quimica, Universidade Estadual de Londrina, Londrina, Pr., Brazil

## Abstract

In the title compound, C_9_H_9_ClN_4_, the dihedral angle between the aromatic rings is 6.25 (9)°. The whole mol­ecule is approximately planar (r.m.s. deviation = 0.070 Å). In the crystal, π–π inter­actions between the centroids of the pyridazine rings [separation = 3.5904 (10) Å] occur.

## Related literature

For background to pyrazolylpyridazine derivatives and for related crystal structures, see: Ather *et al.* (2010**a* ▶,*b* ▶,c* ▶). For hydrogen-bond motifs, see: Bernstein *et al.* (1995 ▶).
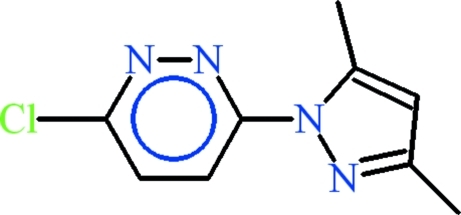


## Experimental

### 

#### Crystal data

C_9_H_9_ClN_4_
*M* *_r_* = 208.65
Monoclinic, 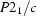

*a* = 11.2773 (3) Å
*b* = 8.4181 (2) Å
*c* = 11.3501 (3) Å
β = 116.529 (1)°
*V* = 964.05 (4) Å^3^
*Z* = 4
Mo *K*α radiation
μ = 0.36 mm^−1^
*T* = 296 K
0.32 × 0.24 × 0.20 mm

#### Data collection

Bruker Kappa APEXII CCD diffractometer
Absorption correction: multi-scan (*SADABS*; Bruker, 2005 ▶) *T*_min_ = 0.903, *T*_max_ = 0.932
6922 measured reflections
1727 independent reflections
1514 reflections with *I* > 2σ(*I*)
*R*_int_ = 0.022

#### Refinement

*R*[*F* ^2^ > 2σ(*F* ^2^)] = 0.031
*wR*(*F* ^2^) = 0.090
*S* = 1.04
1727 reflections
129 parameters
H-atom parameters constrained
Δρ_max_ = 0.16 e Å^−3^
Δρ_min_ = −0.19 e Å^−3^

### 

Data collection: *APEX2* (Bruker, 2009 ▶); cell refinement: *SAINT* (Bruker, 2009 ▶); data reduction: *SAINT*; program(s) used to solve structure: *SHELXS97* (Sheldrick, 2008 ▶); program(s) used to refine structure: *SHELXL97* (Sheldrick, 2008 ▶); molecular graphics: *ORTEP-3* (Farrugia, 1997 ▶) and *PLATON* (Spek, 2009 ▶); software used to prepare material for publication: *WinGX* (Farrugia, 1999 ▶) and *PLATON*.

## Supplementary Material

Crystal structure: contains datablocks global, I. DOI: 10.1107/S1600536810034756/hb5625sup1.cif

Structure factors: contains datablocks I. DOI: 10.1107/S1600536810034756/hb5625Isup2.hkl

Additional supplementary materials:  crystallographic information; 3D view; checkCIF report

## Figures and Tables

**Fig. 1.**
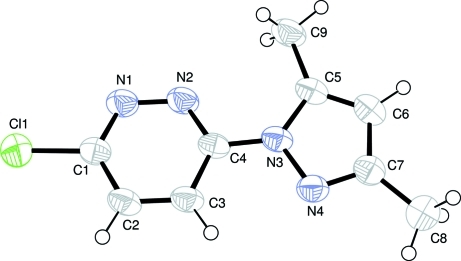
View of the title compound with displacement ellipsoids drawn at the 50% probability level. H-atoms are shown as small spheres of arbitrary radius.

## supplementary crystallographic information

### Comment

In continuation of our studies of pyrazolylpyridazine derivatives (Ather *et al.*, 2010*a*,*b*,*c*), the title compound (I, Fig. 1) is being reported here.

In the title compound, the 3-chloro-pyridazine group A (C1—C4/N1/N2/CL1) and 3,5-dimethyl-pyrazol moiety B (N3/N4/C5—C9) are planar with r. m. s. deviation of 0.0057 and 0.0121 Å, respectively. The dihedral angle between A/B is 6.40 (9)°. The title compound essentially consists of monomers. The molecules are stabilized due to π–π interactions. There exist π–π interactions between the centroids of pyridazine rings at a distance of 3.5904 (10) Å [symmetry code: 1 - *x*, 1 - *y*, 1 - *z*]. The centroids of pyridazine and pyrazol rings are separated at 4.1319 (9) Å [symmetry code: 1 - *x*, 1 - *y*, 1 - *z*] and 4.4233 (9)Å [symmetry code: 1 - *x*, 2 - *y*, 1 - *z*].

### Experimental

3-Chloro-6-hydrazinylpyridazine (1 g, 6.92 mmol) was dissolved in 5 ml of ethanol. To this solution acetylacetone (8 mmol) and acetic acid (0.7 ml) were added and heated for 30 min. The unreacted acetic acid was removed under vacuum and charged to 25 ml of distilled water and filtered. The final product was re-crystallized in ethanol to obtain colourless prisms of (I).

### Refinement

The H-atoms were positioned geometrically (C–H = 0.93–0.96 Å) and were included in the refinement in the riding model approximation, with *U*_iso_(H) = x*U*_eq_(C), where x = 1.5 for methyl and x = 1.2 for aryl H-atoms.

### Crystal data

**Table:**

C_9_H_9_ClN_4_	*F*(000) = 432
*M**_r_* = 208.65	*D*_x_ = 1.438 Mg m^−^^3^
Monoclinic, *P*2_1_/*c*	Mo *K*α radiation, λ = 0.71073 Å
Hall symbol: -P 2ybc	Cell parameters from 1514 reflections
*a* = 11.2773 (3) Å	θ = 3.1–25.3°
*b* = 8.4181 (2) Å	µ = 0.36 mm^−^^1^
*c* = 11.3501 (3) Å	*T* = 296 K
β = 116.529 (1)°	Prism, colourless
*V* = 964.05 (4) Å^3^	0.32 × 0.24 × 0.20 mm
*Z* = 4	

### Data collection

**Table:**

Bruker Kappa APEXII CCD diffractometer	1727 independent reflections
Radiation source: fine-focus sealed tube	1514 reflections with *I* > 2σ(*I*)
graphite	*R*_int_ = 0.022
Detector resolution: 8.10 pixels mm^-1^	θ_max_ = 25.3°, θ_min_ = 3.1°
ω scans	*h* = −13→13
Absorption correction: multi-scan (*SADABS*; Bruker, 2005)	*k* = −10→10
*T*_min_ = 0.903, *T*_max_ = 0.932	*l* = −13→13
6922 measured reflections	

### Refinement

**Table:**

Refinement on *F*^2^	Primary atom site location: structure-invariant direct methods
Least-squares matrix: full	Secondary atom site location: difference Fourier map
*R*[*F*^2^ > 2σ(*F*^2^)] = 0.031	Hydrogen site location: inferred from neighbouring sites
*wR*(*F*^2^) = 0.090	H-atom parameters constrained
*S* = 1.04	*w* = 1/[σ^2^(*F*_o_^2^) + (0.0405*P*)^2^ + 0.3389*P*] where *P* = (*F*_o_^2^ + 2*F*_c_^2^)/3
1727 reflections	(Δ/σ)_max_ < 0.001
129 parameters	Δρ_max_ = 0.16 e Å^−^^3^
0 restraints	Δρ_min_ = −0.19 e Å^−^^3^

### Special details

**Table:**

Geometry. Bond distances, angles *etc*. have been calculated using the rounded fractional coordinates. All su's are estimated from the variances of the (full) variance-covariance matrix. The cell e.s.d.'s are taken into account in the estimation of distances, angles and torsion angles
Refinement. Refinement of *F*^2^ against ALL reflections. The weighted *R*-factor *wR* and goodness of fit *S* are based on *F*^2^, conventional *R*-factors *R* are based on *F*, with *F* set to zero for negative *F*^2^. The threshold expression of *F*^2^ > σ(*F*^2^) is used only for calculating *R*-factors(gt) *etc*. and is not relevant to the choice of reflections for refinement. *R*-factors based on *F*^2^ are statistically about twice as large as those based on *F*, and *R*- factors based on ALL data will be even larger.

### Fractional atomic coordinates and isotropic or equivalent isotropic displacement parameters (Å^2^)

**Table:**

	*x*	*y*	*z*	*U*_iso_*/*U*_eq_	
Cl1	0.76089 (4)	0.97122 (6)	0.60328 (4)	0.0581 (2)	
N1	0.56592 (14)	0.81330 (18)	0.60486 (13)	0.0495 (5)	
N2	0.45739 (14)	0.71964 (18)	0.55421 (13)	0.0487 (5)	
N3	0.29683 (12)	0.57550 (16)	0.38434 (12)	0.0419 (4)	
N4	0.25369 (14)	0.50746 (16)	0.26163 (13)	0.0465 (4)	
C1	0.62272 (15)	0.84978 (19)	0.53015 (15)	0.0427 (5)	
C2	0.58063 (16)	0.7982 (2)	0.40156 (16)	0.0480 (5)	
C3	0.47217 (16)	0.7034 (2)	0.35066 (15)	0.0460 (5)	
C4	0.41180 (14)	0.66847 (18)	0.43182 (14)	0.0389 (5)	
C5	0.21463 (16)	0.5362 (2)	0.44074 (16)	0.0449 (5)	
C6	0.11915 (17)	0.4428 (2)	0.35130 (17)	0.0510 (6)	
C7	0.14674 (16)	0.4269 (2)	0.24272 (16)	0.0464 (5)	
C8	0.07365 (19)	0.3310 (3)	0.12074 (19)	0.0620 (7)	
C9	0.23144 (19)	0.5897 (3)	0.57258 (17)	0.0589 (6)	
H2	0.62480	0.82738	0.35258	0.0576*	
H3	0.43929	0.66322	0.26555	0.0551*	
H6	0.04831	0.39741	0.36007	0.0612*	
H8A	0.11672	0.34089	0.06480	0.0930*	
H8B	−0.01582	0.36889	0.07498	0.0930*	
H8C	0.07304	0.22143	0.14400	0.0930*	
H9A	0.15789	0.55308	0.58622	0.0883*	
H9B	0.23501	0.70358	0.57665	0.0883*	
H9C	0.31225	0.54673	0.63974	0.0883*	

### Atomic displacement parameters (Å^2^)

**Table:**

	*U*^11^	*U*^22^	*U*^33^	*U*^12^	*U*^13^	*U*^23^
Cl1	0.0593 (3)	0.0588 (3)	0.0592 (3)	−0.0085 (2)	0.0291 (2)	−0.0040 (2)
N1	0.0543 (8)	0.0585 (9)	0.0412 (7)	−0.0008 (7)	0.0264 (6)	−0.0005 (6)
N2	0.0513 (8)	0.0624 (9)	0.0396 (7)	−0.0016 (7)	0.0268 (6)	0.0009 (6)
N3	0.0427 (7)	0.0509 (8)	0.0372 (7)	0.0059 (6)	0.0223 (6)	0.0047 (6)
N4	0.0480 (8)	0.0545 (8)	0.0410 (7)	0.0031 (6)	0.0234 (6)	−0.0009 (6)
C1	0.0446 (8)	0.0435 (8)	0.0429 (9)	0.0066 (7)	0.0222 (7)	0.0050 (7)
C2	0.0487 (9)	0.0617 (10)	0.0422 (9)	0.0029 (8)	0.0280 (7)	0.0064 (7)
C3	0.0471 (9)	0.0607 (10)	0.0356 (8)	0.0041 (7)	0.0234 (7)	0.0025 (7)
C4	0.0408 (8)	0.0433 (8)	0.0369 (8)	0.0105 (6)	0.0213 (6)	0.0084 (6)
C5	0.0443 (9)	0.0525 (9)	0.0456 (9)	0.0093 (7)	0.0269 (7)	0.0098 (7)
C6	0.0443 (9)	0.0581 (10)	0.0564 (10)	0.0033 (8)	0.0277 (8)	0.0084 (8)
C7	0.0438 (9)	0.0474 (9)	0.0484 (9)	0.0069 (7)	0.0209 (7)	0.0053 (7)
C8	0.0608 (11)	0.0638 (12)	0.0592 (11)	−0.0047 (9)	0.0249 (9)	−0.0061 (9)
C9	0.0569 (10)	0.0818 (13)	0.0511 (10)	−0.0020 (10)	0.0360 (9)	0.0010 (9)

### Geometric parameters (Å, °)

**Table:**

Cl1—C1	1.7340 (18)	C5—C9	1.492 (3)
N1—N2	1.350 (2)	C6—C7	1.406 (3)
N1—C1	1.307 (2)	C7—C8	1.493 (3)
N2—C4	1.320 (2)	C2—H2	0.9300
N3—N4	1.3781 (18)	C3—H3	0.9300
N3—C4	1.400 (2)	C6—H6	0.9300
N3—C5	1.382 (2)	C8—H8A	0.9600
N4—C7	1.315 (2)	C8—H8B	0.9600
C1—C2	1.388 (2)	C8—H8C	0.9600
C2—C3	1.355 (3)	C9—H9A	0.9600
C3—C4	1.400 (2)	C9—H9B	0.9600
C5—C6	1.353 (2)	C9—H9C	0.9600
			
N2—N1—C1	118.35 (14)	C6—C7—C8	128.16 (18)
N1—N2—C4	119.46 (15)	C1—C2—H2	122.00
N4—N3—C4	118.01 (14)	C3—C2—H2	122.00
N4—N3—C5	111.21 (14)	C2—C3—H3	121.00
C4—N3—C5	130.79 (13)	C4—C3—H3	121.00
N3—N4—C7	105.27 (14)	C5—C6—H6	126.00
Cl1—C1—N1	115.08 (12)	C7—C6—H6	126.00
Cl1—C1—C2	120.01 (14)	C7—C8—H8A	109.00
N1—C1—C2	124.91 (16)	C7—C8—H8B	109.00
C1—C2—C3	116.90 (17)	C7—C8—H8C	109.00
C2—C3—C4	117.07 (15)	H8A—C8—H8B	109.00
N2—C4—N3	116.49 (15)	H8A—C8—H8C	109.00
N2—C4—C3	123.29 (16)	H8B—C8—H8C	109.00
N3—C4—C3	120.22 (13)	C5—C9—H9A	109.00
N3—C5—C6	105.38 (15)	C5—C9—H9B	109.00
N3—C5—C9	125.57 (16)	C5—C9—H9C	109.00
C6—C5—C9	129.05 (19)	H9A—C9—H9B	109.00
C5—C6—C7	107.42 (17)	H9A—C9—H9C	109.00
N4—C7—C6	110.72 (15)	H9B—C9—H9C	109.00
N4—C7—C8	121.10 (17)		
			
C1—N1—N2—C4	−0.2 (2)	C4—N3—C5—C6	−179.65 (16)
N2—N1—C1—Cl1	179.72 (12)	C4—N3—C5—C9	0.8 (3)
N2—N1—C1—C2	−0.6 (3)	N3—N4—C7—C6	0.49 (19)
N1—N2—C4—N3	−178.28 (14)	N3—N4—C7—C8	−177.82 (16)
N1—N2—C4—C3	1.5 (3)	Cl1—C1—C2—C3	179.88 (13)
C4—N3—N4—C7	179.37 (14)	N1—C1—C2—C3	0.2 (3)
C5—N3—N4—C7	−0.21 (18)	C1—C2—C3—C4	0.9 (2)
N4—N3—C4—N2	−173.89 (14)	C2—C3—C4—N2	−1.8 (3)
N4—N3—C4—C3	6.4 (2)	C2—C3—C4—N3	177.92 (15)
C5—N3—C4—N2	5.6 (3)	N3—C5—C6—C7	0.43 (19)
C5—N3—C4—C3	−174.15 (16)	C9—C5—C6—C7	179.98 (19)
N4—N3—C5—C6	−0.15 (19)	C5—C6—C7—N4	−0.6 (2)
N4—N3—C5—C9	−179.73 (17)	C5—C6—C7—C8	177.56 (19)
